# Non-linear association between the dietary index for gut microbiota and the atherogenic index of plasma: insights from a cross-sectional study

**DOI:** 10.3389/fcvm.2025.1556650

**Published:** 2025-07-04

**Authors:** Tian-Ding Liu, Yang-Yang Zheng, Jin-Ying Zhang

**Affiliations:** ^1^ Department of Cardiology, The First Affiliated Hospital of Zhengzhou University, Zhengzhou, China; ^2^ Department of General Surgery, National Center of Gerontology, Beijing Hospital, Institute of Geriatric Medicine, Chinese Academy of Medical Sciences & Peking Union Medical College, Beijing, China

**Keywords:** gut microbiota, dietary index for gut microbiota, cardiovascular disease, atherogenic index of plasma, national health and nutrition examination survey

## Abstract

**Introduction:**

The gut microbiota plays a crucial role in the development of atherosclerosis. The Dietary Index for Gut Microbiota (DI-GM) assesses the impact of diet on gut microbiota, and the Atherogenic Index of Plasma (AIP) serves as a key marker for evaluating atherosclerosis. However, studies exploring the association between DI-GM and AIP remain limited.

**Methods:**

Data from the 2007–2020 National Health and Nutrition Examination Survey (NHANES) were analyzed, including 15,471 participants. Weighted multivariate linear regression models were employed to evaluate the linear association between DI-GM and AIP, while restricted cubic splines (RCS) were used to assess potential nonlinear relationships.

**Results:**

After adjusting for confounding factors, multivariate linear regression analysis demonstrated a significant negative correlation between DI-GM levels and AIP. Each one-point increase in DI-GM was associated with a 0.007-point reduction in AIP (95% CI: −0.012 to −0.002). Categorical analysis further revealed that participants in the DI-GM ≥6 group had significantly lower AIP levels compared to those in lower DI-GM groups (*β* = −0.038, 95% CI: −0.059 to −0.017; P for trend = 0.007). Restricted cubic spline (RCS) analysis identified a significant non-linear dose-response relationship (*P* for non-linearity = 0.018) with a threshold at DI-GM = 3.467. Below this threshold, the association was nonsignificant; however, above it, each unit increase in DI-GM corresponded to a 0.011 decrease in AIP (*P* < 0.001). Subgroup analyses indicated that the relationship between DI-GM and AIP was significantly moderated by age, race/ethnicity, hypertension, and diabetes (*P* for interaction < 0.05).

**Conclusion:**

This study demonstrated a non-linear dose-response relationship between DI-GM levels and AIP, with a significant threshold effect at DI-GM = 3.467. Beyond this threshold, higher DI-GM levels were linked to lower AIP, moderated by age, race/ethnicity, hypertension, and diabetes.

## 1 Introduction

Atherosclerosis, characterized by the accumulation of plaques within arterial walls, is a key pathological factor in the development of cardiovascular disease (CVD) (1, 2). In recent years, the atherogenic index of plasma (AIP) has gained significant attention for its critical role in predicting atherosclerosis and CVD risk (3). AIP is calculated as the logarithmic ratio of triglycerides to high-density lipoprotein cholesterol (HDL-C) and reflects the particle size and esterification rate of low-density lipoprotein cholesterol (LDL-C), which are closely associated with lipoprotein lipase activity (4, 5). Moreover, studies have demonstrated that AIP is not only an independent and superior predictor of atherosclerosis compared to traditional lipid parameters but also serves as a potential biomarker for evaluating the severity of CVD (6, 7).

Meanwhile, the pivotal role of gut microbiota in the pathogenesis of CVD has garnered increasing interest (8–10). Advances in high-throughput gene sequencing technologies have further elucidated the intricate relationship between gut microbiota and cardiovascular health (11). Changes in the composition and functionality of gut microbiota have been strongly linked to the onset and progression of atherosclerosis (11). Notably, dietary interventions cause significant changes in the diversity, balance, and function of gut microbiota, which opens up a new strategy for the prevention and treatment of CVD. For instance, dietary patterns rich in anti-inflammatory foods, such as fruits, vegetables, and whole grains, have been associated with healthier gut microbiota profiles, improved lipid metabolism, and reduced cardiovascular risk (12).

To systematically evaluate the impact of diet on gut microbiota, researchers have developed the Dietary Index for Gut Microbiota (DI-GM), a scoring system comprising 10 beneficial and 4 detrimental dietary components (13). This index assesses how dietary patterns influence gut microbiota health and provides a foundation for optimizing diets to prevent and manage diseases (14). Despite its potential, the relationship between DI-GM and AIP remains underexplored.

This study aims to address this knowledge gap by investigating the association between DI-GM and AIP and examining the potential factors influencing this relationship. By Utilizing data from the National Health and Nutrition Examination Survey (NHANES) spanning 2007–2020, we seek to provide comprehensive and robust evidence supporting the role of dietary patterns in the prevention of atherosclerosis.

## 2 Method

### 2.1 Study population

**Figure 1:**
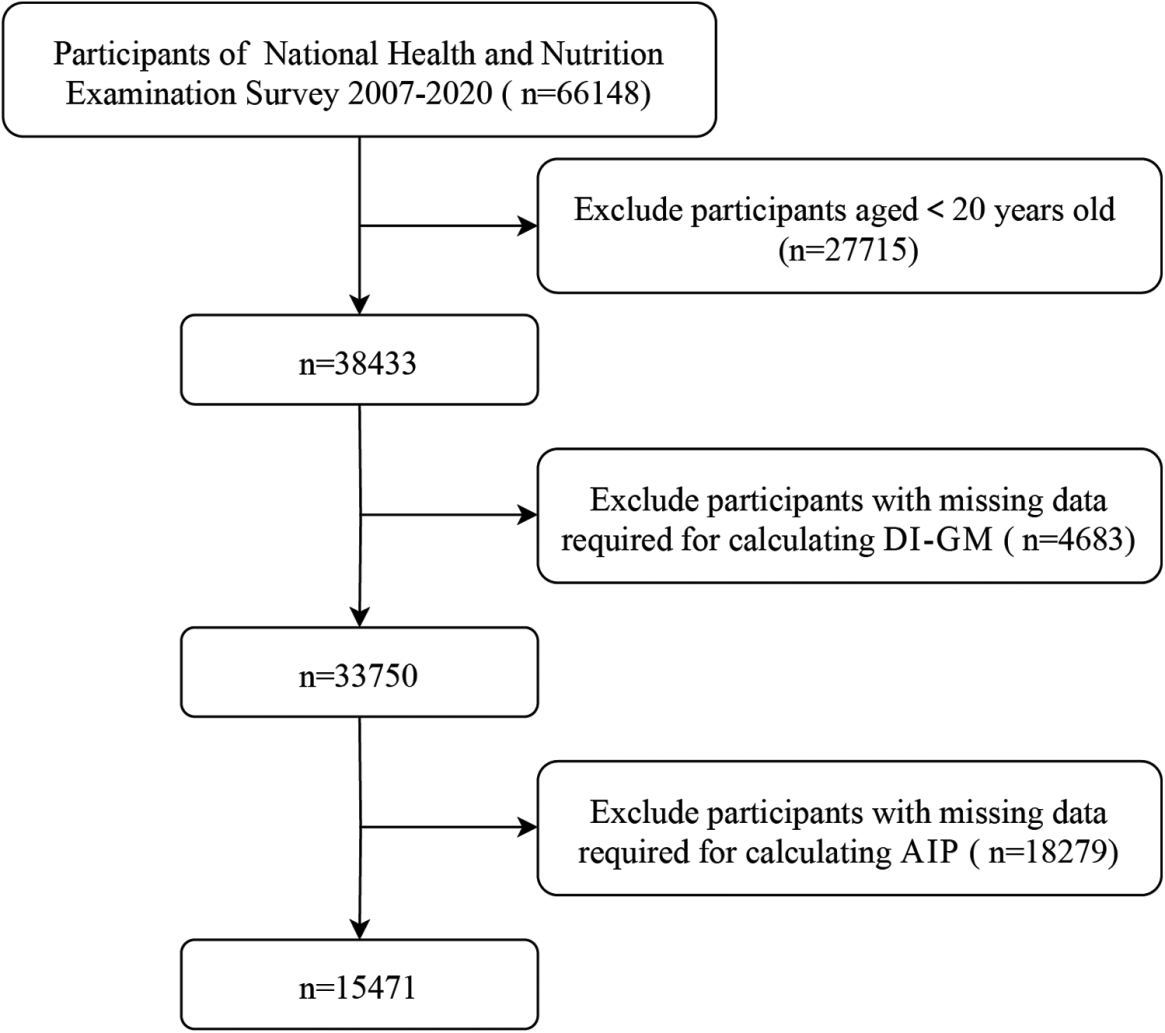
Selection of participants in the study.

This study analyzed data from the 2007–2020 NHANES. NHANES employs a multi-cycle, cross-sectional design and advanced sampling techniques to recruit a nationally representative cohort. 66,148 participants provided informed written consent before participating in the survey, 27,715 participants were excluded because they were younger than 20 years old, 4,683 participants were excluded for missing data required for calculating DI-GM and 18,279 participants were exclude for missing data required for calculating AIP (as detailed in Figure 1). The final analysis included data from 15,471 U.S. adults aged 20 years and older.

### 2.2 Calculation of AIP

The exposure variable, AIP, as log10(TG/HDL-C), was first established as a biomarker of plasma atherosclerosis by Dobiasova M, Frohlich J (15). Direct immunoassay or precipitation methods measured HDL-C levels following CDC standardized procedures. Each subject had fasting venous blood drawn for TG measurement.

### 2.3 Calculation of Di-GM

This study used the scoring system developed by Kase et al. to calculate the DI-GM, based on 14 foods or nutrients (13). The DI-GM was calculated using data from two 24 h dietary recalls, which is shown in Supplementary Table S1. Beneficial foods received a score of 1 if intake met or exceeded the sex-specific median, otherwise 0. Unfavorable foods received a score of 0 if intake met or exceeded the sex-specific median (or above 40% energy from fat), otherwise 1. The total DI-GM score spanned a range of 0 to 14, with beneficial foods contributing 0–10 points and unfavorable foods 0–4 points. Participants were divided into four groups based on quartiles of total scores: 0–3, 4, 5, and 6 or higher.

### 2.4 Covariates

Sociodemographic and socioeconomic characteristics included age (continuous), gender (male, and female), race (Non-Hispanic Black, Non-Hispanic White, Mexican American, and Other Race), marital status (never married/living with partner, married, and divorced/separated/widowed), education level (less than high school graduate, high school graduate or GED, and some college or above), and poverty income ratio [classified as low income (<1.30), middle income (1.30–3.49), and high income (≥3.50)]. Health behaviors included smoking status (never, ex-smoker, and current-smoker), alcohol intake (no-drinking, and drinking). Other potential confounders were diabetes (no/yes), and hypertension (no/yes). The NHANES dataset includes comprehensive information on demographics, diet, examinations, laboratory results, and questionnaires, along with detailed tools, methods, usage guidelines, and FAQs, as outlined in the NHANES manuals and reports.

### 2.5 Statistical analysis

This study excluded samples with missing information on AIP or dietary recall data. Samples with missing covariate values exceeding 20% were not included in the analysis. For covariates with less than 20% missing data, the “mice” package in R was employed to perform multiple imputations. Participant demographic and clinical characteristics were categorized by quartiles of DI-GM. The relationships between categorical variables, presented as frequencies and weighted percentages, were assessed using the Rao-Scott *χ*² test. Continuous variables were reported as weighted means and their corresponding standard errors (SE). The Wilcoxon rank-sum test for complex survey samples was employed to evaluate differences in continuous variables.

Weighted multivariable linear regression models were applied to evaluate the linear association between DI-GM (as both a continuous and categorical variable) and AIP. Model 1 included unadjusted data. Model 2 adjusted for age, gender, and race/ethnicity. Model 3 further included adjustments for marital status, education level, and poverty-to-income ratio (PIR). Finally, Model 4 accounted for additional factors such as alcohol intake, smoking status, hypertension, and diabetes, alongside all covariates included in the previous models. We employed Restricted cubic splines (RCS) regression models to further explore the dose–response between DI-GM and AIP, adjusting for all confounding variables in model 4 (16). The Akaike Information Criterion guided the location and number of knots in RCS knots to balance model fit and overfitting. If a nonlinear relationship was confirmed, a threshold effect analysis was performed using a two-segment linear regression model. This approach analyzed the association between DI-GM and AIP separately on either side of the inflection point, providing deeper insights into the nature of the relationship.

Subgroup analyses were conducted to identify factors influencing the relationship between DI-GM and AIP. The analysis involved stratifying the final analytical sample by age (<65, and ≥65 years), gender (male and female), race (Non-Hispanic White, Non-Hispanic Black, Mexican American, and Other Race), marital status (married/living with partner, never married, and widowed/divorced/separated), education level (less than high school graduate, high school graduate or GED, and some college or above), poverty income ratio [classified as low income (<1.30), middle income (1.30–3.49), and high income (≥3.50)], smoking status (never, ex-smoker, and current-smoker), alcohol intake (no-drinking, and drinking), diabetes(no/yes), hypertension(no/yes), respectively. A multiplicative interaction term among the subgroups, DI-GM, and AIP was fitted into the model to assess for potential interaction effects.

All analyses were performed using R version 4.4.1 (R Foundation for Statistical Computing, Vienna, Austria http://www.R-project.org). Differences with *P* < 0.05 indicated statistical significance.

## 3 Results

### 3.1 Baseline characteristics of participants

The characteristics of the participants categorized by DI-GM groups was presented in Table 1. A total of 15,471 individuals participated in the study with an average (SE) age of 48.32 (0.28) years, with an average (SE) poverty income ratio of 2.94 (0.04) and a mean (SE) AIP of −0.06 (0.01). Participants in the highest DI-GM group (Q4) were significantly more likely to be older, female, of non-Hispanic White backgrounds, married, have higher educational attainment, higher PIR, be never-smokers, consume alcohol, and not have diabetes (all *P* < 0.05) compared to those in the lower DI-GM groups. Notably, participants with higher DI-GM levels also exhibited significantly lower AIP values (*P* < 0.001).

**Table 1:** Characteristics of the NHANES 2007–2020 participants based on quartiles of DI-GM.

Characteristics	Total adults (*N* = 15,471)	DIGM Q1 (*N* = 3,837)	DIGM Q2 (*N* = 3,896)	DIGM Q3 (*N* = 3,543)	DIGM Q4 (*N* = 4,195)	*P* value
Age, mean (SE), years	48.32 (0.28)	46.47 (0.48)	47.17 (0.43)	48.08 (0.40)	50.77 (0.47)	<0.001
Age, *n* (%)						0.001
20–65	11,742 (79.81)	3,011 (82.41)	3,006 (81.13)	2,693 (79.46)	3,032 (77.12)	
≥65	3,729 (20.19)	826 (17.59)	890 (18.87)	850 (20.54)	1,163 (22.88)	
Gender, *n* (%)						0.003
Male	7,515 (48.25)	1,993 (50.44)	1,912 (50.54)	1,692 (46.74)	1,918 (45.97)	
Female	7,956 (51.75)	1,844 (49.56)	1,984 (49.46)	1,851 (53.26)	2,277 (54.03)	
Race and Ethnicity, *n* (%)						<0.001
Non-Hispanic White	6,461 (66.57)	1,465 (61.97)	1,479 (62.21)	1,485 (66.98)	2,032 (73.12)	
Non-Hispanic Black	3,225 (10.74)	1,036 (15.52)	882 (12.38)	695 (9.77)	612 (6.62)	
Mexican American	2,319 (8.45)	555 (9.08)	643 (10.25)	577 (9.20)	544 (5.98)	
Other Race	3,466 (14.24)	781 (13.43)	892 (15.16)	786 (14.04)	1,007 (14.28)	
Marital status, *n* (%)						<0.001
Married/Living with partner	9,243 (62.76)	2,213 (59.07)	2,224 (59.77)	2,159 (63.11)	2,647 (67.59)	
Never married	2,807 (18.47)	808 (21.45)	770 (19.75)	629 (19.77)	600 (14.27)	
Widowed/Divorced/Separated	3,421 (18.77)	816 (19.48)	902 (20.48)	755 (17.12)	948 (18.14)	
Education level, *n* (%)						<0.001
Less than high school graduate	3,707 (15.60)	990 (17.74)	1,103 (20.28)	835 (14.99)	779 (10.78)	
High school graduate or GED	3,519 (23.32)	1,075 (29.12)	889 (24.67)	774 (21.56)	781 (19.28)	
Some college or above	8,245 (61.08)	1,772 (53.15)	1,904 (55.05)	1,934 (63.46)	2,635 (69.94)	
PIR, *n* (%)	2.94 (0.04)	2.67 (0.05)	2.73 (0.05)	3.00 (0.05)	3.27 (0.05)	<0.001
PIR, *n* (%)						<0.001
≤1.3	4,988 (22.90)	1,384 (26.31)	1,420 (27.32)	1,126 (23.11)	1,058 (16.71)	
1.3–3.5	5,857 (35.29)	1,560 (39.64)	1,477 (36.61)	1,295 (32.97)	1,525 (32.77)	
>3.5	4,626 (41.82)	893 (34.05)	999 (36.07)	1,122 (43.92)	1,612 (50.52)	
Smoking, *n* (%)						<0.001
Never	8,577 (54.68)	2,000 (53.55)	2,147 (54.18)	1,997 (56.02)	2,433 (54.88)	
Ex-smoker	3,800 (26.06)	906 (23.80)	901 (24.62)	845 (24.44)	1,148 (30.09)	
Current-smoker	3,094 (19.27)	931 (22.64)	848 (21.20)	701 (19.54)	614 (15.03)	
Drinking, *n* (%)						0.040
No	2,151 (10.79)	487 (10.27)	577 (12.31)	495 (11.03)	592 (9.80)	
Yes	13,320 (89.21)	3,350 (89.73)	3,319 (87.69)	3,048 (88.97)	3,603 (90.20)	
AIP, mean (SE)	−0.06 (0.01)	−0.05 (0.01)	−0.04 (0.01)	−0.06 (0.01)	−0.09 (0.01)	<0.001
Hypertension, *n* (%)						0.220
No	8,772 (60.93)	2,117 (59.65)	2,199 (60.24)	2,045 (63.00)	2,411 (60.87)	
Yes	6,699 (39.07)	1,720 (40.35)	1,697 (39.76)	1,498 (37.00)	1,784 (39.13)	
Diabetes, *n* (%)						<0.001
No	12,092 (83.04)	2,885 (80.45)	3,014 (82.01)	2,822 (84.34)	3,371 (84.79)	
Yes	3,379 (16.96)	952 (19.55)	882 (17.99)	721 (15.66)	824 (15.21)	

All means and SEs for continuous variables and percentages for categorical variables were weighted. The DI-GM ranges from 0 to 13 [including beneficial to gut microbiota [ranges from 0 to 9] and unfavorable to gut microbiota [ranges from 0 to 4]] and grouped according to 0–4, 5, 6, and >6.

AIP, atherogenic index of plasma; DI-GM, dietary index for gut microbiota; NHANES, national health and nutrition examination survey; PIR, poverty income ratio; SE, standard error.

### 3.2 Associations between DI-GM and AIP

All four models exhibited a negative correlation between DI-GM and AIP (Table 2). In model 1, after adjusting for no covariates, each 1-point increase in DI-GM was associated with a decrease of 0.011 in AIP. Model 2 (95% CI: −0.017, −0.006), additionally adjusted for age, gender, and race/ethnicity, showed that this negative relationship persisted as significant (*β* = −0.013, 95% CI: −0.018, −0.008). In Model 3, further adjustments were made for marital status, education level, and poverty income ratio (PIR), with the association persisting (*β* = −0.010, 95% CI: −0.015, −0.005). In Model 4, additional adjustments for smoking status, alcohol intake hypertension, and diabetes, retained the significant inverse relationship between DI-GM and AIP (*β* = −0.007, 95% CI: −0.012, −0.002).

**Table 2:** Association between DI-GM and AIP of the NHANES 2007–2020 participants.

Variables	Model 1	*P* value	Model 2	*P* value	Model 3	*P* value	Model 4	*P* value
	β (95% CI)		β (95% CI)		β (95% CI)		β (95% CI)	
DI-GM	−0.011 (−0.017, −0.006)	<0.001	−0.013 (−0.018, −0.008)	<0.001	−0.01 (−0.015, −0.005)	<0.001	−0.007 (−0.012, −0.002)	0.007
DI-GM group								
Q1	1 [Reference]		1 [Reference]		1 [Reference]		1 [Reference]	
Q2	0.011 (−0.008, 0.031)	0.261	0.004 (−0.015, 0.024)	0.650	0.005 (−0.014, 0.024)	0.596	0.005 (−0.014, 0.024)	0.592
Q3	−0.015 (−0.038, 0.008)	0.187	−0.021 (−0.043, 0.001)	0.064	−0.012 (−0.033, 0.009)	0.244	−0.013 (−0.033, 0.008)	0.232
Q4	−0.042 (−0.064, −0.020)	<0.001	−0.052 (−0.074, −0.031)	<0.001	−0.039 (−0.060, −0.018)	<0.001	−0.038 (−0.059, −0.017)	<0.001
Trend test		<0.001		<0.001		<0.001		0.007

AIP, atherogenic index of plasma; DI-GM, dietary index for gut microbiota; CI, confidence interval; PIR, poverty income ratio.

Crude: unadjusted model.

Model 1 no covariates were adjusted.

Model 2 was adjusted for age, gender, and race/ethnicity.

Model 3 was adjusted for age, gender, race/ethnicity, marital status, education level, and PIR.

Model 4 was adjusted for age, gender, race/ethnicity, marital status, education level, PIR, alcohol intake, and smoking status, hypertension, and diabetes.

For categorical analysis, DI-GM levels were divided into four groups: 0–3, 4, 5, and 6 or higher, with 0–3 serving as the reference group. In Model 1, after adjusting for no covariates, DI-GM of 6 or higher were significantly correlated with a decrease of 0.042 in AIP (95% CI: −0.064, −0.020; *P* for trend <0.001). Model 2, with additional adjustment for age, gender, and race/ethnicity, confirmed this significant association (*β* = −0.052, 95% CI: −0.074, −0.031; *P* for trend <0.001). In Model 3, further adjusted for marital status, education level, and PIR, the significant inverse association persisted (*β* = −0.039, 95% CI: −0.060, −0.018; *P* for trend <0.001). Model 4, which additionally adjusted for smoking status, alcohol intake hypertension, and diabetes, also sustained the significant inverse relationship between DI-GM of 6 or higher and reduced AIP (*β* = −0.038, 95% CI: −0.059, −0.017; *P* for trend = 0.007).

### 3.3 Dose-response analysis of DI-GM with AIP

**Figure 2:**
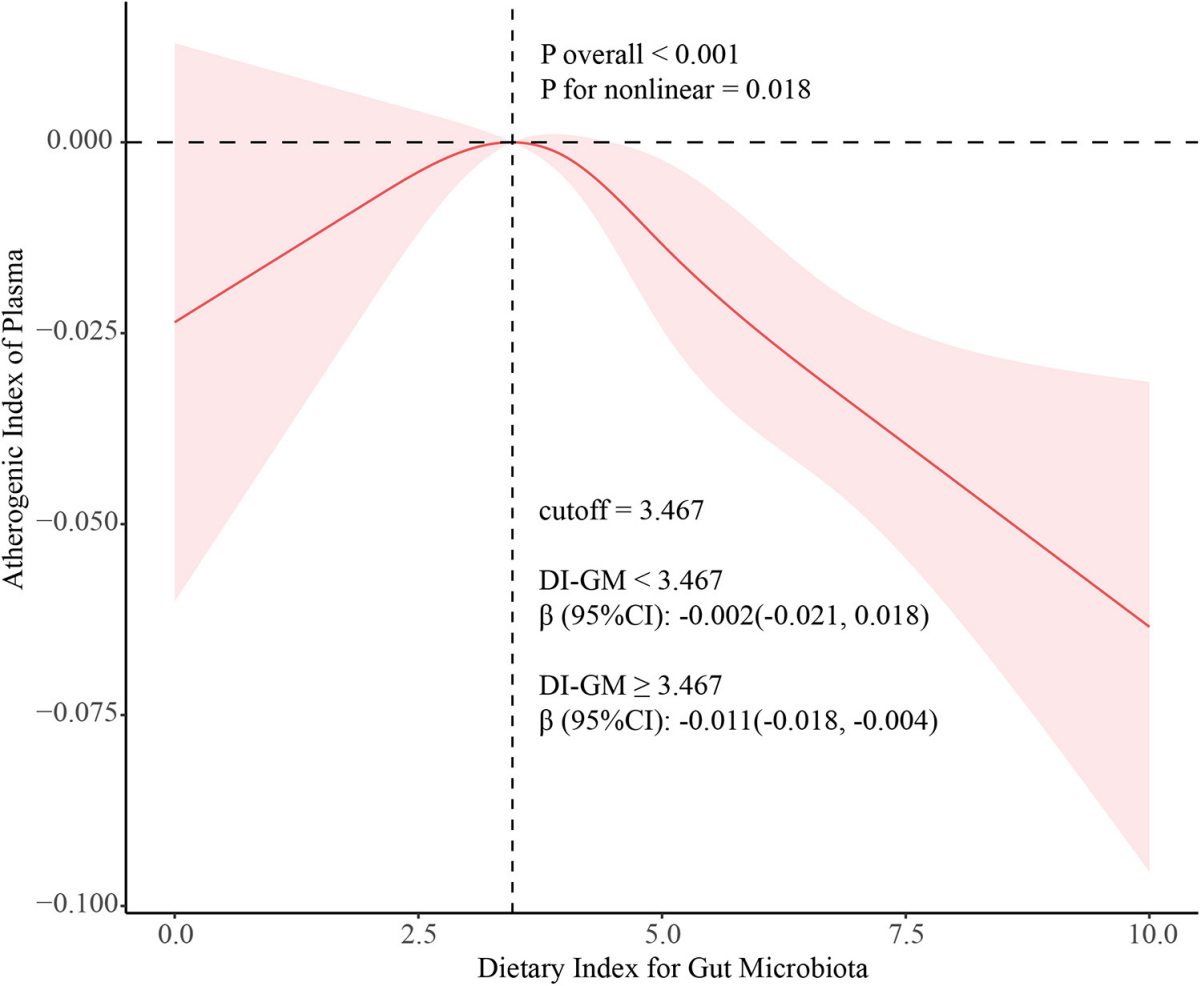
Association between DI-GM and MAFLD using a restricted cubic spline model. Multivariable adjusted odds ratios (solid line) with 95% confidence interval (shaded area) for the association of DI-GM with MAFLD disease. Adjusted for age (continuous), gender (male, and female), race (Non-Hispanic White, Non-Hispanic Black, Mexican American, and Other Race), marital status (married/living with partner, never married, and widowed/divorced/separated), education level (less than high school graduate, high school graduate or GED, and some college or above), PIR (continuous), smoking status (never, ex-smoker, and current-smoker), alcohol intake (no-drinking, and drinking). DI-GM, dietary index for gut microbiota; MAFLD, metabolic associated fatty liver disease; PIR, poverty income ratio.

A multivariable-adjusted restricted cubic spline analysis revealed a significant non-linear dose-response relationship between DI-GM and AIP (*P* for non-linearity = 0.018; *P* for overall < 0.001), with a critical inflection point at DI-GM = 3.467 (Figure 2). Further two-piecewise linear regression analysis, adjusted for potential confounders such as age, gender, race/ethnicity, marital status, education level, poverty-to-income ratio, alcohol intake, smoking status, hypertension, and diabetes, demonstrated distinct trends (Table 3). When DI-GM was below 3.467, the relationship with AIP was not significant (*β* = −0.002, 95% CI: −0.021 to 0.018; *P* = 0.872). However, when DI-GM was ≥3.467, each unit increase in DI-GM was associated with a significant 0.011 decrease in AIP (*β* = −0.011, 95% CI: −0.018 to −0.004; *P* < 0.001), highlighting a non-linear association and a potential threshold effect.

**Table 3:** The results of two-piecewise linear regression analysis between DI-GM (cutoff = 3.467) and AIP of the NHANES 2007–2020 participants.

Variables	Model 1	*P* value	Model 2	*P* value	Model 3	*P* value	Model 4	*P* value
	β (95% CI)		β (95% CI)		β (95% CI)		β (95% CI)	
DI-GM < 3.467	0.001 (−0.019, 0.020)	0.936	−0.004 (−0.023, 0.015)	0.681	−0.004 (−0.023, 0.016)	0.719	−0.002 (−0.021, 0.018)	0.872
DI-GM ≥ 3.467	−0.018 (−0.026, −0.010)	<0.001	−0.019 (−0.027, −0.011)	<0.001	−0.014 (−0.021, −0.006)	<0.001	−0.011 (−0.018, −0.004)	<0.001
*P* for log likelihood ratio test		<0.001		<0.001		<0.001		<0.001

AIP, atherogenic index of plasma; DI-GM, dietary index for gut microbiota; CI, confidence interval; PIR, poverty income ratio.

Crude: unadjusted model.

Model 1 no covariates were adjusted.

Model 2 was adjusted for age, gender, and race/ethnicity.

Model 3 was adjusted for age, gender, race/ethnicity, marital status, education level, and PIR.

Model 4 was adjusted for age, gender, race/ethnicity, marital status, education level, PIR, alcohol intake, and smoking status, hypertension, and diabetes.

### 3.4 Subgroup analyses

Subgroup analyses were conducted to examine whether any factors modified the relationship between DI-GM and AIP (Table 4). After adjusting for confounders, no significant interactions were found across subgroups stratified by gender, marital status, education level, PIR, smoking status, or alcohol intake (*P* for interaction > 0.05). Conversely, age, race/ethnicity, hypertension, and diabetes emerged as a potential moderating factor in the relationship between DI-GM and AIP (*P* for interaction < 0.05).

**Table 4:** Associations between DI-GM and AIP of the NHANES 2007–2020 participants, stratified by selected factors.

Character	DI-GM < 3.467			DI-GM ≥ 3.467		
	OR (95% CI)	*P* value	*P* for interaction	OR (95% CI)	*P* value	*P* for interaction
Age, years			0.287			0.047
20–65	−0.006 (−0.028, 0.016)	0.601		−0.015 (−0.024, −0.007)	<0.001	
≥65	0.017 (−0.017, 0.051)	0.316		−0.001 (−0.014, 0.012)	0.868	
Gender			0.678			0.517
Male	−0.006 (−0.033, 0.022)	0.685		−0.011 (−0.021, 0.000)	0.047	
Female	0.003 (−0.027, 0.033)	0.854		−0.011 (−0.020, −0.001)	0.027	
Race and Ethnicity			0.844			0.011
Non-Hispanic White	−0.006 (−0.033, 0.022)	0.692		−0.015 (−0.025, −0.005)	0.003	
Non-Hispanic Black	−0.005 (−0.039, 0.029)	0.765		−0.002 (−0.016, 0.011)	0.719	
Mexican American	0.019 (−0.029, 0.068)	0.427		−0.010 (−0.025, 0.004)	0.160	
Other Race	0.003 (−0.052, 0.058)	0.915		0.003 (−0.008, 0.014)	0.557	
Marital status			0.667			0.877
Married/Living with partner	0.000 (−0.027, 0.026)	0.983		−0.011 (−0.021, −0.001)	0.037	
Never married	−0.014 (−0.058, 0.030)	0.525		−0.017 (−0.034, −0.001)	0.038	
Widowed/Divorced/Separated	0.011 (−0.033, 0.054)	0.625		−0.006 (−0.020, 0.007)	0.328	
Education level			0.676			0.533
Less than high school graduate	−0.002 (−0.043, 0.038)	0.912		−0.003 (−0.021, 0.015)	0.736	
High school graduate or GED	0.005 (−0.027, 0.037)	0.768		−0.015 (−0.035, 0.005)	0.151	
Some college or above	−0.009 (−0.035, 0.018)	0.512		−0.010 (−0.019, −0.001)	0.026	
PIR			0.202			0.124
≤1.3	0.021 (−0.012, 0.053)	0.205		−0.024 (−0.037, −0.012)	<0.001	
1.3–3.5	−0.013 (−0.041, 0.014)	0.334		−0.012 (−0.023, −0.001)	0.027	
>3.5	0.004 (−0.031, 0.040)	0.804		−0.004 (−0.014, 0.006)	0.435	
Smoking			0.518			0.315
Never	−0.007 (−0.037, 0.022)	0.610		−0.010 (−0.019, −0.001)	0.023	
Ex-smoker	−0.004 (−0.041, 0.033)	0.819		−0.014 (−0.029, 0.000)	0.049	
Current-smoker	0.018 (−0.017, 0.054)	0.310		−0.002 (−0.020, 0.016)	0.858	
Drinking			0.071			0.460
No	−0.032 (−0.072, 0.008)	0.111		−0.002 (−0.018, 0.014)	0.801	
Yes	0.004 (−0.016, 0.024)	0.714		−0.012 (−0.020, −0.004)	0.005	
Hypertension			0.912			<0.001
No	−0.001 (−0.025, 0.023)	0.916		−0.018 (−0.027, −0.010)	<0.001	
Yes	−0.001 (−0.030, 0.027)	0.935		0.002 (−0.007, 0.011)	0.689	
Diabetes			0.017			0.023
No	−0.012 (−0.034, 0.010)	0.286		−0.015 (−0.022, −0.007)	<0.001	
Yes	0.03 (−0.005, 0.066)	0.094		0.007 (−0.009, 0.023)	0.390	

Each stratification was adjusted for age, gender, race/ethnicity, marital status, education level, PIR, smoking status, alcohol intake, hypertension, and diabetes, if not already stratified.

AIP, atherogenic index of plasma; CI, confidence interval; DI-GM, dietary index for gut microbiota; NHANES, national health and nutrition examination survey; PIR, poverty income ratio.

### 3.5 Sensitivity analysis

Sensitivity analysis was conducted to ensure the robustness of the findings. Additional adjustments for total caloric intake and overall dietary quality were performed. The results remained highly consistent, and the sensitivity analyses further confirmed the substantial association between DI-GM and AIP (Supplementary Table S2).

## 4 Discussion

With the accelerating pace of population aging, CVD—a major age-related condition—has become the leading cause of death and disability worldwide, highlighting the urgent need for effective prevention and intervention strategies (17). Among various risk factors, dyslipidemia plays a central role in the development of CVD. Although clinical interventions have long focused on lowering LDL-C to reduce CVD risk, this single marker does not fully capture the complexity of lipid metabolic disorders (18, 19). Other indicators, such as the apolipoprotein B to apolipoprotein A-I ratio (ApoB/ApoA-I) and coronary artery calcium scores, may provide more accurate assessments of atherosclerosis severity, but their clinical utility remains limited due to high testing costs and low accessibility (20, 21). AIP, calculated as log₁₀(TG/HDL-C), has emerged as a promising biomarker that reflects the dynamic balance between pro-atherogenic and anti-atherogenic lipids (15). Studies have demonstrated that AIP outperforms conventional lipid markers in predicting adverse cardiovascular events (22, 23). Moreover, AIP independently predicts the presence of vulnerable plaques beyond traditional factors (24). Targeting AIP may thus represent a potential strategy for improving the lipoprotein profile and reducing the risk of cardiovascular events.

Recent advances in high-throughput gene sequencing have revealed the critical role of gut microbiota in CVD (25). Alterations in the composition and function of the gut microbiome have been closely associated with atherosclerosis and heart failure (26, 27). A healthy diet is essential for cardiovascular risk management, exerting both direct effects on cardiovascular health and indirect effects through modulation of the gut microbiota. To assess dietary quality, several scoring systems have been widely adopted, including the Healthy Eating Index (HEI), the Alternate Healthy Eating Index (AHEI), the Mediterranean Diet Score (MDS), and the Dietary Approaches to Stop Hypertension (DASH) (28, 29). Although validated for predicting cardiovascular outcomes, these indices do not explicitly incorporate microbiota-related dietary components, and their associations with gut microbial diversity and composition remain inconsistent (29–32).

In response to this gap, more targeted indices have emerged. For instance, the Sulfate-Metabolizing Diet Score reflects dietary patterns associated with sulfur-metabolizing bacteria, which are linked to increased colorectal cancer risk; however, its applicability is limited to specific microbial taxa (33). In contrast, the DI-GM provides a more comprehensive framework for evaluating diet–microbiome interactions (13). Though originally designed to assess microbiota-related dietary quality, DI-GM also correlates positively with HEI-2015 and MDS, supporting its dual relevance in both microbiome and overall dietary health research (13).

In this cross-sectional study, we found a significant inverse association between DI-GM and AIP, suggesting that a higher DI-GM score may exert a protective effect against atherosclerosis and cardiovascular diseases. Interestingly, we observed a non-linear dose–response relationship between DI-GM and AIP, with a significant threshold effect identified at a DI-GM value of 3.467. When DI-GM was below this threshold, its association with AIP was not statistically significant; however, beyond this point, higher DI-GM scores were progressively associated with lower AIP values. Practically, a DI-GM of 3.467 typically reflects dietary patterns characterized by only limited intake of microbiota-supportive foods and/or continued consumption of adverse components. These findings suggest that a minimum dietary quality may be required to exert beneficial effects on lipid metabolism. At lower DI-GM levels, individuals may remain in a state of gut dysbiosis, and further improvements in DI-GM may be necessary to elicit favorable lipid profiles. Our results underscore that only when dietary patterns achieve a certain degree of “gut-friendliness” can the diet–microbiota–cardiometabolic axis be effectively activated to improve lipid metabolism.

Several biological mechanisms may account for the inverse association observed above the DI-GM threshold. First, higher DI-GM diets promote the growth of short-chain fatty acid (SCFA)-producing bacteria (13, 34). SCFAs such as acetate, propionate, and butyrate have been shown to exert multiple lipid-modulating effects, including enhancing HDL cholesterol and reducing triglycerides, thereby improving AIP (35). In addition, trimethylamine N-oxide (TMAO) production may also play a role. Low DI-GM diets, which are often rich in animal-derived foods (e.g., red and processed meats), supply abundant precursors such as carnitine and choline, which gut microbes convert into TMA and subsequently oxidize into TMAO—a metabolite known to promote atherosclerosis by inhibiting cholesterol efflux, promoting foam cell formation, and enhancing inflammation and thrombosis (36, 37). In contrast, high DI-GM diets, predominantly plant-based, not only reduce TMAO precursors but also reshape the gut microbiota toward a composition less conducive to TMAO production. Therefore, reduced TMAO levels at higher DI-GM scores may contribute to lower AIP. Furthermore, several favorable components of DI-GM—such as avocados, soybeans, dietary fiber, and whole grains—help reduce intestinal cholesterol absorption (38–41). Antioxidant-rich foods, including cranberries, broccoli, green tea, and coffee, exert antioxidant and anti-inflammatory effects, which may further decrease cardiovascular risk (42–45). Chickpeas and fermented dairy products promote the growth of probiotics, thereby improving insulin sensitivity (46, 47). Conversely, unfavorable components such as high-fat diets and refined grains can stimulate the production of pro-inflammatory metabolites like lipopolysaccharides, which contribute to chronic inflammation and plaque instability (48, 49). Collectively, high DI-GM dietary patterns may improve AIP and reduce cardiovascular risk by modulating gut microbiota composition, microbial metabolites, and host inflammatory responses.

These findings suggest that higher DI-GM scores may exert a protective effect against atherosclerosis and cardiovascular diseases. Our findings highlight a critical inflection point in the diet–microbiota–host metabolism axis, suggesting that only after achieving a sufficient level of dietary quality can the gut microbiota exert their full potential to favorably regulate host lipid metabolism. This has important implications for precision nutrition strategies aimed at reducing cardiometabolic risk through gut microbiota-targeted dietary interventions. If confirmed by future longitudinal studies, then dietary recommendations based on DI-GM could be used to individualize dietary interventions aimed at enhancing lipid profiles and preventing CVD.

This study has several limitations. First, because this work is cross-sectional, we cannot determine if changes in DI-GM lead to changes in AIP. Longitudinal studies and randomized controlled trials are required to establish whether dietary interventions that target DI-GM can decrease AIP. Second, the use of self-reported data may have introduced recall bias, potentially affecting the accuracy of the collected data. Third, dietary information in the NHANES dataset was collected using only two 24 h dietary recalls, which may not adequately capture temporal variations in dietary patterns over time. Future research should employ repeated dietary assessments at multiple time points to better evaluate the dynamic relationship between DI-GM and AIP. In addition, although we controlled for many demographic and clinical variables, we did not account for potential confounders such as macronutrient distribution, other lipid markers, and physical activity directly. Future analyses with these variables included may help to better understand the relationships observed. Moreover, although the DI-GM specifically emphasizes gut microbiota–related dietary components, future research should investigate whether it provides additional predictive value for cardiovascular health beyond established dietary indices such as the MDS and the DASH diet. Lastly, as the study population was drawn from the NHANES, the generalizability of the findings to other populations and regions remains uncertain.

## 5 Conclusion

In summary, this study demonstrated a significant inverse association between DI-GM and AIP, characterized by a non-linear dose-response relationship with a critical threshold at DI-GM = 3.467. Above this threshold, higher DI-GM levels were strongly associated with lower AIP, with the most pronounced reductions observed in the DI-GM ≥6 group. Subgroup analyses revealed that this relationship was moderated by age, race/ethnicity, hypertension, and diabetes. These findings highlight the potential of DI-GM as a dietary marker for cardiovascular risk and emphasize the importance of personalized dietary strategies.

## Data Availability

The original contributions presented in the study are included in the article/Supplementary Material, further inquiries can be directed to the corresponding author.
