# Supplementary material for: Non-linear association between the dietary index for gut microbiota and the atherogenic index of plasma: insights from a cross-sectional study
Source: Front Cardiovasc Med. 2025 Jul 4;12:1556650. doi: 10.3389/fcvm.2025.1556650 (PMC12271143; doi:10.3389/fcvm.2025.1556650)
Supplement: Supplementary file 1 [file Datasheet1.docx]

**Supplementary Table 1.** Components and scoring criteria of DI-GM in NHANES.

| Components of DI-GM | Included Foods within the Component | Scoring criteria |
| --- | --- | --- |
| Beneficial to gut microbiota  (10 Components) | Avocados | Each component was scored as follows: 1 – if consumption was at or above the sex-specific median; 0 – otherwise. |
|  | Broccoli |  |
|  | Chickpeas |  |
|  | Coffee |  |
|  | Cranberries |  |
|  | Fermented dairy (including yogurt, cheese, kefir, sour cream, buttermilk) |  |
|  | Fiber |  |
|  | Green tea |  |
|  | Soybean (including Soy milk, Tofu) |  |
|  | Whole grains (grains defined as whole grains, containing the entire grain kernel—the bran, germ, and endosperm) |  |
| Unfavorable to gut microbiota  (4 Components) | Processed meat (including frankfurters, sausages, corned beef, and luncheon meat that are made from beef, pork, or poultry) | Each component was scored as follows: 0 – if consumption was at or above the sex-specific median; 1 – otherwise. |
|  | Red meat (including beef, veal, pork, lamb, and game meat; excludes organ meat and cured meat) |  |
|  | Refined grains (refined grains that do not contain all of the components of the entire grain kernel) |  |
|  | High-fat diet (% energy) | Scoring criteria: 0 – if consumption was at or above 40% of total energy intake; 1 – otherwise. |

Abbreviations: DI-GM, dietary index for gut microbiota; NHANES, National Health and Nutrition Examination Survey.

**Supplementary Table 2.** Associations between DI-GM and AIP of the NHANES 2007-2020 participants, stratified by selected factors.

| character | DI-GM < 3.467 | | |  | DI-GM≥3.467 | | |
| --- | --- | --- | --- | --- | --- | --- | --- |
|  | OR (95% CI) | P value | p for interaction |  | OR (95% CI) | P value | p for interaction |
| Age, years |  |  | 0.933 |  |  |  | 0.039 |
| 20-65 | 0.005(-0.020, 0.029) | 0.697 |  |  | -0.019(-0.027, -0.010) | <0.001 |  |
| ≥65 | 0.009(-0.033, 0.051) | 0.656 |  |  | -0.001(-0.015, 0.013) | 0.904 |  |
| Gender |  |  | 0.276 |  |  |  | 0.845 |
| Male | -0.010(-0.042, 0.022) | 0.537 |  |  | -0.015(-0.026, -0.003) | 0.017 |  |
| Female | 0.021(-0.014, 0.055) | 0.240 |  |  | -0.012(-0.021, -0.003) | 0.011 |  |
| Race and Ethnicity |  |  | 0.720 |  |  |  | 0.028 |
| Non-Hispanic White | -0.003(-0.035, 0.030) | 0.870 |  |  | -0.017(-0.026, -0.008) | <0.001 |  |
| Non-Hispanic Black | 0.017(-0.014, 0.049) | 0.268 |  |  | -0.004(-0.019, 0.011) | 0.613 |  |
| Mexican American | 0.034(-0.012, 0.080) | 0.141 |  |  | -0.015(-0.033, 0.003) | 0.092 |  |
| Other Race | 0.019(-0.045, 0.083) | 0.555 |  |  | 0.000(-0.015, 0.014) | 0.966 |  |
| Marital status |  |  | 0.276 |  |  |  | 0.523 |
| Married/Living with partner | 0.015(-0.017, 0.048) | 0.349 |  |  | -0.013(-0.024, -0.002) | 0.020 |  |
| Never married | -0.031(-0.081, 0.020) | 0.231 |  |  | -0.023(-0.042, -0.004) | 0.017 |  |
| Widowed/Divorced/Separated | 0.013(-0.033, 0.058) | 0.574 |  |  | -0.005(-0.018, 0.007) | 0.376 |  |
| Education level |  |  | 0.915 |  |  |  | 0.050 |
| Less than high school graduate | -0.002(-0.051, 0.046) | 0.928 |  |  | 0.011(-0.008, 0.030) | 0.247 |  |
| High school graduate or GED | 0.004(-0.031, 0.039) | 0.821 |  |  | -0.020(-0.038, -0.002) | 0.028 |  |
| Some college or above | 0.003(-0.031, 0.036) | 0.867 |  |  | -0.006(-0.017, 0.004) | 0.226 |  |
| PIR |  |  | 0.393 |  |  |  | 0.131 |
| ≤1.3 | 0.027(-0.010, 0.064) | 0.152 |  |  | -0.018(-0.031, -0.005) | 0.009 |  |
| 1.3∼3.5 | -0.002(-0.037, 0.032) | 0.892 |  |  | -0.023(-0.036, -0.010) | <0.001 |  |
| >3.5 | 0.007(-0.034, 0.049) | 0.720 |  |  | -0.004(-0.014, 0.006) | 0.433 |  |
| Smoking |  |  | 0.780 |  |  |  | 0.108 |
| Never | -0.002(-0.029, 0.025) | 0.873 |  |  | -0.018(-0.027, -0.009) | <0.001 |  |
| Ex-smoker | 0.027(-0.025, 0.078) | 0.302 |  |  | -0.012(-0.025, 0.001) | 0.077 |  |
| Current-smoker | 0.020(-0.020, 0.061) | 0.311 |  |  | -0.003(-0.018, 0.011) | 0.662 |  |
| Drinking |  |  | 0.126 |  |  |  | 0.874 |
| no | -0.031(-0.080, 0.018) | 0.213 |  |  | -0.008(-0.027, 0.010) | 0.374 |  |
| yes | 0.011(-0.013, 0.035) | 0.357 |  |  | -0.014(-0.022, -0.006) | <0.001 |  |
| Hypertension |  |  | 0.219 |  |  |  | 0.023 |
| no | 0.016(-0.011, 0.042) | 0.245 |  |  | -0.020(-0.029, -0.011) | <0.001 |  |
| yes | -0.009(-0.038, 0.021) | 0.570 |  |  | -0.005(-0.014, 0.004) | 0.284 |  |
| Diabetes |  |  | 0.128 |  |  |  | 0.046 |
| no | -0.004(-0.028, 0.021) | 0.756 |  |  | -0.018(-0.025, -0.010) | <0.001 |  |
| yes | 0.024(-0.018, 0.066) | 0.254 |  |  | 0.004(-0.015, 0.022) | 0.686 |  |

Each stratification was adjusted for age, gender, race/ethnicity, marital status, education level, PIR, smoking status, alcohol intake, hypertension, diabetes, total caloric intake, and dietary quality (HEI 2015) if not already stratified.

Abbreviations: AIP, Atherogenic index of plasma; CI, confidence interval; DI-GM, dietary index for gut microbiota; NHANES, National Health and Nutrition Examination Survey; PIR, Poverty Income Ratio; HEI, Healthy Eating Index.
